# Efficacy and safety of montelukast for pediatric obstructive sleep apnea syndrome: A protocol for systematic review and meta-analysis

**DOI:** 10.1097/MD.0000000000023958

**Published:** 2021-01-22

**Authors:** Jun-Li Bao, Xinyuan-Gao, Yu-Bo Han, Ke Zhang, Li Liu

**Affiliations:** a Heilongjiang University of Chinese Medicine, No.24, Heping Road, Xiangfang District, Haerbin; b Fist Affiliated Hospital, Heilongjiang University of Chinese Medicine, Haerbin, Heilongjiang, China.

**Keywords:** meta-analysis, montelukast, pediatric obstructive sleep apnea syndrome

## Abstract

**Background:**

Pediatric obstructive sleep apnea syndrome (OSAS) is significant public concern. Clinical practice indicates that montelukast has certain therapeutic advantages, while there is a lack of evidence-based medicine support. The aim of this study is to synthesize related data to explore efficacy and safety of montelukast for pediatric OSAS.

**Methods:**

Data in Pubmed, EMBASE, CENTRAL, CBM, CNKI, WanFang, VIP databases were comprehensively searched. All the randomized controlled trials (RCTs) in OSAS children were identified, in which the effects of montelukast on a range of outcomes were compared. The search had a deadline of January 1, 2020. Two investigators independently conducted data extraction and assessed the literature quality of the included studies. The Revman5.3 software was used for meta-analysis of the included literature.

**Results:**

The efficacy and safety of montelukast in the treatment of pediatric OSAS were evaluated in terms of apnea hypopnea index (AHI), the Pittsburgh Sleep Quality Index, the Epworth Sleep Scale (ESS), neck circumference, important index in Polysomnography: sleep efficiency, desaturation index, total sleep time.

**Conclusions:**

This study provides reliable evidence-based support for the clinical application of montelukast in the treatment of pediatric OSAS.

**PROSPERO registration number:**

CRD42020146940.

## 1 Introduction

Obstructive sleep apnea syndrome (OSAS) in children refers to a series of clinical syndromes caused by partial collapse or complete obstruction of upper airway during sleep, which disturbs normal ventilation and sleep structure during sleep.^[1]^ OSAS is characterized by snoring, mouth opening, restless sleep, enuresis, hyperhidrosis and inattention.^[2]^ In severe cases, OSAS can also lead to growth retardation,^[3]^ cognitive dysfunction,^[4]^ adenoid facial features^[5]^ and cardiovascular diseases.^[6]^ Adenoid hypertrophy or tonsillar hypertrophy is the main cause of OSAS in children.^[7]^ Surgical removal of adenoids and/or tonsils is the first choice for the treatment of childhood OSAS without contraindications.^[8]^ However, there are still a considerable number of OSAS children with adenoidectomy and/or tonsillectomy sleep apnea can not be completely improved.^[9]^ These has instigated exploration of nonsurgical therapeutic alternatives.

Previous studies demonstrated the abundant expressions of leukotrienes and their receptors in adenotonsillar tissues, which lead to the therapeutic use of leukotriene receptor antagonist such as montelukast in children who have mild OSAS.^[10]^ At present, several studies reported the significant benefits of montelukast in alleviating OSAS severity.^[11]^ However, it is not all children who responded to the treatment, restricting of the promotion of this method to a certain extent.^[12]^ Therefore, this study aimed to systematically assess the efficacy and safety of montelukast in the treatment of OSAS and provide a reliable reference for the clinical application of montelukast in the treatment of OSAS.

## 2 Methods

### 2.1 Protocol register

This protocol of systematic review and meta-analysis has been drafted under the guidance of the preferred reporting items for systematic reviews and meta-analysis protocols (PRISMA-P).^[13]^ Moreover, it has been registered on the PROSPERO (registration number: CRD42020146940).

### 2.2 Ethics

Since this is a protocol with no patient recruitment and personal information collection, approval by the ethics committee is not required.

### 2.3 Eligibility criteria

#### 2.3.1 Types of studies

We will collect all available randomized controlled trials (RCTs) on montelukast in the treatment of OSAS, regardless of publication status, and region, but language will be restricted to Chinese and English.

#### 2.3.2 Patients

The patients (1–14 years old) were definitively diagnosed with OSAS in accordance with the polysomnography (PSG) with no limitation of nationality, race, sex, course of disease, etc.

#### 2.3.3 Intervention

Patients in the treatment group received oral montelukast, while patients in the control group received placebo. There were no restrictions on the dose, frequency, and treatment course of montelukast.

#### 2.3.4 Outcome indicators

1. Primary outcome: Apnea hypopnea index (AHI) an index that indicate the severity of sleep apnea. It is presented by a count of the number of apneas and hypopneas per hour of sleep.^[14]^
2. Secondary outcomes: the Pittsburgh Sleep Quality Index,^[15]^ the Epworth Sleep Scale (ESS),^[16]^ neck circumference,^[17]^ important index in Polysomnography: sleep efficiency, desaturation index, total sleep time.^[18]^

### 2.4 Exclusion criteria

1. Animal experiments, reviews, case reports, and repetitive publications or search outcomes
2. The published papers were abstracts or the data were incomplete and the papers with complete data were not available after contacting the author.
3. Papers containing less than 10 cases;
4. Papers assessed as high risk of bias by randomization or concealed distribution.^[19]^
5. Papers with no relevant outcome indicators.

### 2.5 Information sources

The search will use a sensitive subject and topic-based strategy from inception to January 1, 2020. Databases searched will be PubMed, EMBASE, the Cochrane Central Register of Controlled Trials (CENTRAL), Chinese Biomedical Literature Database (CBM), China National Knowledge Infrastructure (CNKI), Wan-Fang Database and China Science and Technology Journal Database (VIP).

### 2.6 Search strategy

Take “montelukast (meng lu si te)”, “obstructive sleep apnea syndrome (zu sai xing shui mian hu xi zan ting zong he zheng)”, “snoring (da han)” as the Chinese search terms and search in Chinese databases, including CNKI, Wanfang, VIP. Take “montelukast”, “montelukast sodium”, “obstructive sleep apnea syndrome”, “Obstructive Sleep Apneas”, “Obstructive Sleep Apnea” as the English terms and search terms and search in English databases, including PubMed, EMBASE, CENTRAL, CBM were searched manually. Taking PubMed as an example, the retrieval strategy is shown in Table 1.

**Table 1:** Search strategy for the PubMed database.

#1	montelukast
#2	montelukast sodium
#3	Singulair
#4	MK 0476
#5	Sodium 1-(((1-(3-(2-(7-chloro-2-quinolinyl)ethenyl)phenyl)-3-(2-(1-hydroxy-1-methylethyl)phenyl)propyl)thio)methyl)cyclopropylacetate)
#6	Leukotriene Antagonists
#7	#1 OR #2 OR #3 OR #4 OR#5 OR #6
#8	obstructive sleep apnea syndrome
#9	Apneas, Obstructive Sleep) OR Obstructive Sleep Apneas
#10	Sleep Apneas, Obstructive
#11	Obstructive Sleep Apnea
#12	OSAHS
#13	Syndrome, Sleep Apnea, Obstructive
#14	Sleep Apnea Syndrome, Obstructive
#15	Apnea, Obstructive Sleep
#16	Sleep Apnea Hypopnea Syndrome
#17	Syndrome, Obstructive Sleep Apnea
#18	Upper Airway Resistance Sleep Apnea Syndrome
#19	Syndrome, Upper Airway Resistance, Sleep Apnea
#20	#8 OR #9 OR #10 OR #11 OR #12 OR #13 OR #14 OR #15 OR #16 OR #17 OR #18 OR #19
#21	#7 AND #20

### 2.7 Data filtering and extraction

**Figure 1:**
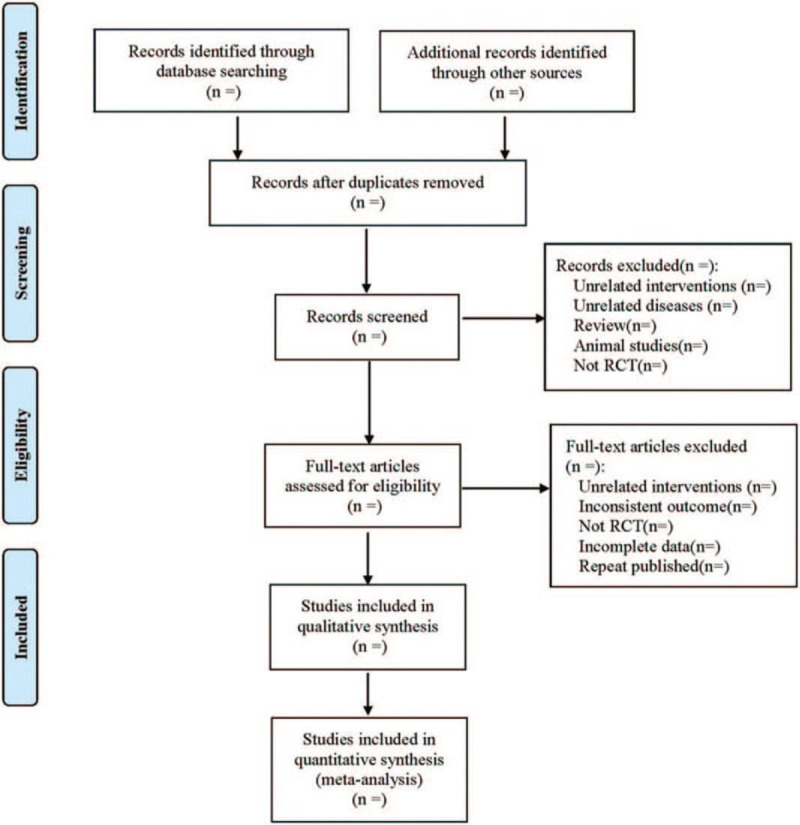
The process of literature filtering.

Referring to the method of research selection in version 5.0 of the Cochrane Collaboration Network System Evaluator Manual, according to the preferred reporting items for systematic reviews and meta-analysis (PRISMA) flow chart, 2 authors used the EndNote V.X9 document management software to independently screen and check the literature according to the above inclusion and exclusion criteria, and check each other. A third reviewer reviewed the extracted data and stored the original data in a secure computer to avoid additional changes. At the same time, Excel 2016 was used to extract relevant information, including: The variables, namely, article title, author(s), journal title, year of publication, sample size, sex, age, interventions, the duration of treatment, and outcome. The process of literature filtering is shown in Figure 1.

### 2.8 Literature quality assessment

The quality of all included studies was assessed by 2 authors. The AMSTAR measurement tool was adopted, consisting of 16 problems.^[20]^

1. Did the research questions and inclusion criteria for the review include the components of PICO?
2. Did the report of the review contain an explicit statement that the review methods were established prior to the conduct of the review and did the report justify any significant deviations from the protocol?
3. Did the review authors explain their selection of the study designs for inclusion in the review?
4. Did the review authors use a comprehensive literature search strategy?
5. Did the review authors perform study selection in duplicate?
6. Did the review authors perform data extraction in duplicate?
7. Did the review authors provide a list of excluded studies and justify the exclusions?
8. Did the review authors describe the included studies in adequate detail?
9. Did the review authors use a satisfactory technique for assessing the risk of bias (RoB) in individual studies that were included in the review?
10. Did the review authors report on the sources of funding for the studies included in the review?
11. If meta-analysis was performed, did the review authors use appropriate methods for statistical combination of results?
12. If meta-analysis was performed, did the review authors assess the potential impact of RoB in individual studies on the results of the meta-analysis or other evidence synthesis?
13. Did the review authors account for RoB in primary studies when interpreting/discussing the results of the review?
14. Did the review authors provide a satisfactory explanation for, and discussion of, any heterogeneity observed in the results of the review?
15. If they performed quantitative synthesis did the review authors carry out an adequate investigation of publication bias (small study bias) and discuss its likely impact on the results of the review?
16. Did the review authors report any potential sources of conflict of interest, including any funding they received for conducting the review?

Discrepancy was resolved through the discussion between the 2 reviewers. Judgments were independently made by 2 investigators; disagreements were remedied after the discussion with a third investigator.

### 2.9 Statistical analysis

#### 2.9.1 Data analysis and processing

A meta-analysis was conducted using RevMan 5.3 only when enough and suitable data were harvested. In terms of dichotomous variable, the results were calculated as risk ratios (RR). For continuous variable, mean differences (MD) were adopted. Both of the mentioned elements corresponding to 95% confidence interval (CI) were calculated. Heterogeneity was ascertained using the *I*^*2*^. *I*^*2*^ < 50% revealed that the studies exhibited homogeneity, so fixed effects model was used; otherwise, the random effects model was used. In the presence of heterogeneity, sensitivity analyses would be conducted to investigate heterogeneity sources.

#### 2.9.2 Dealing with missing data

If there are missing data in the article, contact the author via email for additional information. If the author cannot be contacted, or the author has lost relevant data, descriptive analysis will be conducted instead of meta-analysis.

#### 2.9.3 Sensitivity analysis

In order to test the stability of meta-analysis results of indicators, a one-by-one elimination method will be adopted for sensitivity analysis.

#### 2.9.4 Assessment of reporting biases

Publication bias was assessed by funnel plot and Egger test. Egger test was performed for no less than 10 studies, whereas the trim-and-fill analysis was conducted in the presence of publication bias.

#### 2.9.5 Evidence quality evaluation

The AMSTAR measurement tool will be used to assess the quality of evidence. The quality of evidence will be rated as high, moderate, low, and very low.

## 3 Discussion

OSAS is a common and highly prevalent disorder in the pediatric age range.^[21]^ OSAS imposes a vast array of morbidities including neurocognitive, behavioral, cardiovascular, and metabolic.^[22]^ Pediatric OSAS is usually treated by adenotonsillectomy. However, a substantial proportion of children with OSAS undergoing adenotonsillectomy may develop postoperative complications and have persistent disease after surgery.^[23]^ In recent years, nonsurgical therapeutic alternatives has been explored actively.^[24]^ Some studies suggested that montelukast could be effective.^[25]^ This study aimed to systematically assess the efficacy and safety of montelukast in the treatment of pediatric OSAS and provide a reliable reference for the clinical application of montelukast in the treatment of pediatric OSAS.

However, this systematic review had some limitations: there were differences in the doses of montelukast used in the included studies and the condition of the patients disease. There may be some clinical heterogeneity. The course of disease was also different, which may have affected the results to some extent. Constrained by language ability, we only searched English and Chinese literature and may ignore studies or reports in other languages.

## Author contributions

**Conceptualization:** Junli Bao.

**Data curation:** Xinyuan Gao.

**Funding acquisition:** Li Liu.

**Investigation:** Yubo Han.

**Methodology:** Ke Zhang.

**Project administration:** Junli Bao.

**Software:** Junli Bao, Xinyuan Gao.

**Supervision:** Yubo Han.

**Validation:** Xinyuan Gao.

**Writing – original draft:** Junli Bao, Xinyuan Gao.

**Writing – review & editing:** Junli Bao, Li Liu.
